# Extended parental provisioning and variation in vertebrate brain sizes

**DOI:** 10.1371/journal.pbio.3002016

**Published:** 2023-02-28

**Authors:** Carel P. van Schaik, Zitan Song, Caroline Schuppli, Szymon M. Drobniak, Sandra A. Heldstab, Michael Griesser

**Affiliations:** 1 Department of Ecology of Animal Societies, Max Planck Institute for Animal Behavior, Konstanz, Germany; 2 Department of Evolutionary Anthropology, University of Zurich, Zurich, Switzerland; 3 Department of Evolutionary Biology and Environmental Studies, University of Zurich, Zurich, Switzerland; 4 Development and Evolution of Cognition Research group, Max Planck Institute for Animal Behavior, Konstanz, Germany; 5 Evolution & Ecology Research Centre, School of Biological, Environmental & Earth Sciences, University of New South Wales, Sydney, Australia; 6 Institute of Environmental Sciences; Jagiellonian University, Krakow, Poland; 7 Department of Biology, University of Konstanz, Konstanz, Germany; 8 Center for the Advanced Study of Collective Behavior, University of Konstanz, Konstanz, Germany; 9 Department of Collective Behavior, Max Planck Institute of Animal Behavior, Konstanz, Germany

## Abstract

Large brains provide adaptive cognitive benefits but require unusually high, near-constant energy inputs and become fully functional well after their growth is completed. Consequently, young of most larger-brained endotherms should not be able to independently support the growth and development of their own brains. This paradox is solved if the evolution of extended parental provisioning facilitated brain size evolution. Comparative studies indeed show that extended parental provisioning coevolved with brain size and that it may improve immature survival. The major role of extended parental provisioning supports the idea that the ability to sustain the costs of brains limited brain size evolution.

## Abstract 2

How do large-brained species account for the high energy requirements of their growing brains when they cannot yet produce the cognitive benefits needed to provide the energy? In this Essay, the authors suggest that extended parental provisioning could solve this energy dilemma.

## Introduction: Expensive brains

**Fig 1:**
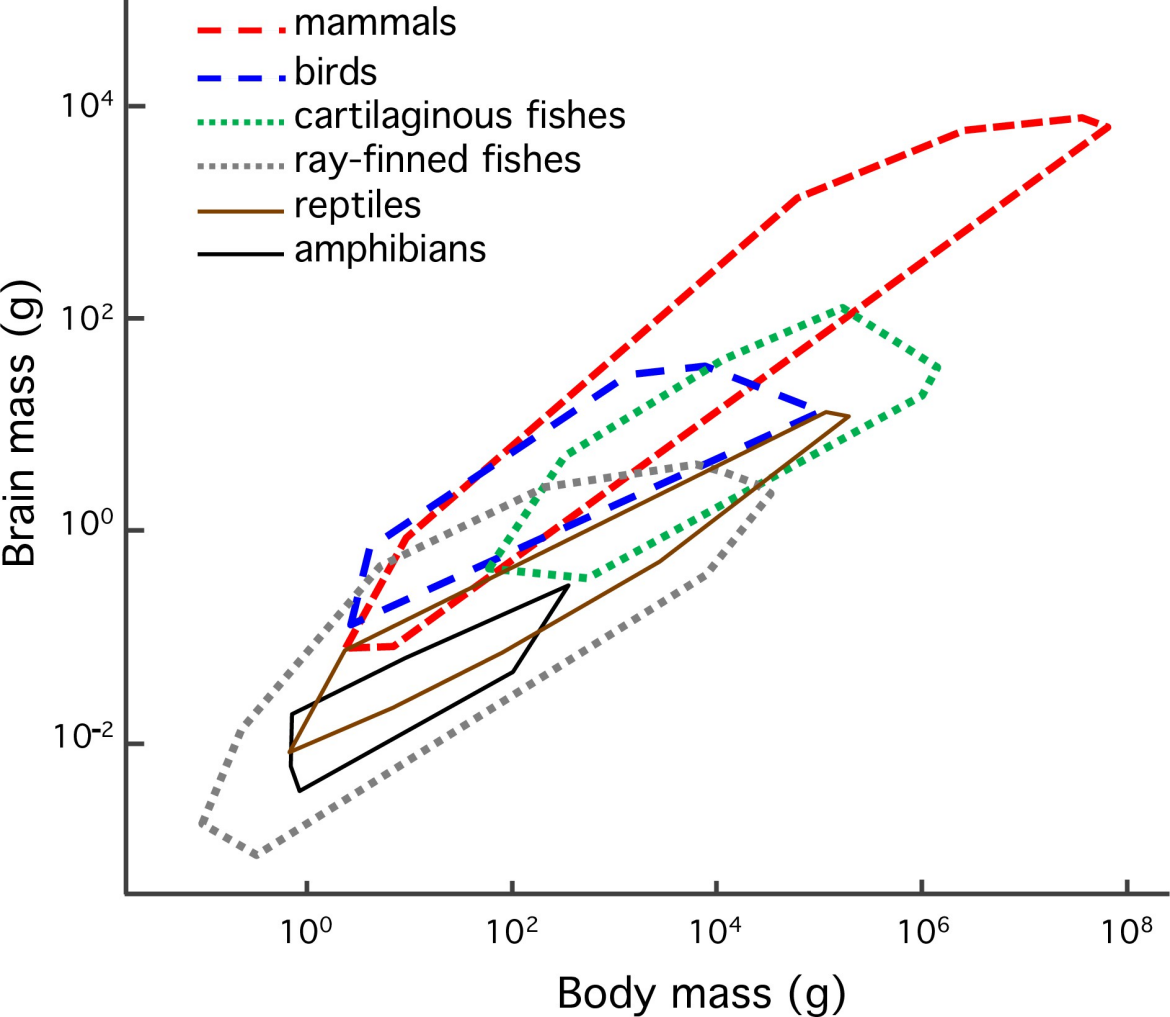
Brain size-body size envelopes of the major vertebrate lineages, to illustrate both intra-lineage and inter-lineage variation. The long-dashed outlines represent the 2 endothermic lineages (birds and mammals), the dotted outlines represent fishes, and the solid outlines the 2 ectothermic tetrapod lineages (amphibians and reptiles). Redrawn after [97].

The brain analyzes and integrates the inputs from our senses, regulates our physiology, and generates the motor commands for our movements. In addition, it is responsible for everything between perception and action, i.e., cognition. Relative to body size, brain size is extremely variable across vertebrates [1,2]. Mean brain sizes of ectothermic (fishes, amphibians, and reptiles) and endothermic (birds and mammals) lineages differ about 10-fold, but also vary considerably within each lineage (Fig 1). In addition, brains have tended to become larger over evolutionary time [1,2]. Understanding this striking variation and these evolutionary trends in relative brain size is a major task for comparative biology.

Brain size is generally positively correlated with the amount of sensory information a species processes (e.g., electrosensing in mormyroid fishes: [3]; stereoscopic vision in primates: [4,5]) or the precision of its motor control (e.g., number of legs in lizards: [6]; manipulation complexity in primates: [7]), suggesting that these enhanced sensorimotor functions alone may explain brain size changes without reference to greater cognitive abilities [8]. Nonetheless, comparative studies also show a clear link between relative brain size and more narrowly defined cognitive abilities, such as greater capacity for independent or social learning [9–12], and thus greater domain-general intelligence [13,14] and executive functions, such as self-control [15,16]. Because sensorimotor capacities do not vary systematically within species, intraspecific correlations between brain size and domain-general intelligence would provide even more convincing evidence for an effect of brain size on narrow-sense cognition. Indeed, in humans, brain size explains a modest, but robust proportion of variation in intelligence [17,18], a result now replicated in chimpanzees [19] and chestnut-headed thrushes [20].

These 3 sets of abilities (i.e., sensory input, cognitive processing, and motor output) are expected to coevolve. Having perfect information without sophisticated cognitive processing and advanced abilities to act upon the world would not be adaptive. Brain size should therefore also predict behavioral performance in fitness-enhancing activities. Indeed, larger-brained species are capable of extractive foraging [21] and tend to be more innovative in the foraging domain (primates: [22]; birds: [23]). They are also better at avoiding predators (mammals: [24]; birds: [25]) and more likely to survive when introduced into novel areas by humans (mammals: [26]; birds: [27]; reptiles and amphibians: [28]). These effects could ultimately lead to correlated evolution between brain size and maximum lifespan. Comparative studies have confirmed such a correlation for most mammals [29–31], birds [32,33], and frogs [34], though not for reptiles [35]. An improved ability to form social bonds with conspecifics may also be linked to larger brain size [36] (but see [37]). A more indirect consequence of improved survival is that larger-brained species have more stable populations (primates: [38]; birds: [39]), and hence, a reduced risk of local extinction [40].

All these findings indicate that increasing brain size should very often be adaptive, as confirmed by the upward evolutionary trend in brain size [1]. One might therefore expect that, once controlled for body size differences, brain sizes would be similar across taxa. However, this is not the case: major differences between closely related lineages exist [41], as do differences between more distantly related lineages with similar cognitive demands, such as between social carnivores and anthropoid primates [42]. These differences imply that some brain-size related costs prevent the evolution of similar brain sizes in particular lineages, despite these various cognitive benefits [43]. Thus, a comprehensive explanation for the variation in brain size requires that we incorporate the fitness costs of increased brain (cf. [43]). This is what the expensive brain hypothesis [30] attempts to do.

Brains are unusually costly organs due to their high energy use per unit weight [44–46] and especially because energy allocation to the brain cannot not be down-regulated during times of starvation (brain sparing: [47,48]). Interruption of this constant energy flow to the brain generally has lasting negative consequences for brain development and cognitive performance [49–52]. As a result, brain size is presumably limited by the organism’s ability to sustain the energy turnover needed to grow or maintain the brain in response to cognitive opportunities in the ecological or social environment. Reduced energy inputs can arise due to ecologically imposed limitations on overall energy acquisition, in particular, the inability to adequately deal with periods of unavoidable food scarcity. Alternatively, reduced allocation to other competing costly functions, such as digestion [45] or production (i.e., growth and reproduction [29–34,53–55]), could enable increases in brain sizes. A recent review [56] found extensive empirical support for this hypothesis. In sum, the expensive brain hypothesis helps to explain why brain size does not always correspond to expectations based on the cognitive demands and opportunities offered by social systems or ecological niches. This conclusion holds even if controlled for taxonomic variation in neuron densities [57,58].

**Fig 2:**
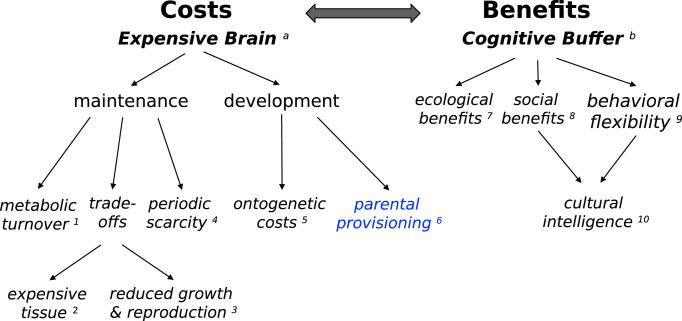
Categorization of the various hypotheses to explain evolutionary variation in relative brain size among vertebrates. Two complementary clusters of hypotheses focus on either costs (expensive brain) or benefits (cognitive buffer). The brain size of a given species should reflect the balance between all relevant processes. In this review, we elaborate the developmental aspects of the expensive brain hypothesis. Key references are provided in superscripts: a: [124] (see also delayed benefits: [125]; b: [30]; 1: metabolic demands: [126,127]; 2: [45]; 3: [29, 30]; 4: [128,129]; 5: [55] (see also maturational constraints and brain malnutrition risks: [125]); 5: [83,88,108] (also: maternal energy); 7: [130,131]; 8: [132,133]; 9: [22]; 10: [134].

Because the expensive brain hypothesis focuses on the costs of brain size, it complements the various hypotheses postulating benefits to larger brain size (Fig 2). The 2 categories of hypotheses are therefore not exclusive, although the strength of each may vary taxonomically.

## The expensive brain: Developmental aspects

Whereas most previous work on the expensive brain hypothesis focused on the consequences of the high energy costs for adults, here we focus on its developmental aspects (Fig 2). The high energy demands of growing brains create various problems.

First, brains are unusual organs in that they must acquire their cognitive and motor functions through practice and learning and therefore perform poorly before they are fully grown and differentiated. In most mammals, brain growth is largely completed around weaning [59,60], whereas adulthood is postponed until bodily growth is completed. Accordingly, many species tend to reach adult-level ecological skills such as the recognition of the values of specific food items and basic processing techniques around weaning [61–63], while they master most complex skills later: extractive foraging [64] and especially tool use [65]. Birds differ greatly from mammals in that both brain and body growth are completed very early [66], well (sometimes years [67]) before reproduction starts. This suggests that the time needed for acquiring ecological skills (e.g., food processing [62,68] or predator recognition [69,70]) limits the age at which adulthood is reached. Overall, therefore, immatures in most birds and many mammals are ecologically less competent than adults, and some undergo a long phase of practice and learning before reaching adult skill levels (birds: [71,72]; mammals: [61,62]), even after brain growth has been completed.

Second, immature birds and especially mammals are in a phase of high ecological risk for 2 main reasons. They are less experienced and often smaller, which exposes them to higher risk of predation or disease [64,70]. They are also generally socially subordinate to adults, and thus may be peripheralized, either socially or in terms of habitat quality. They consequently face particularly high mortality risks, especially at higher population densities [73,74]. These 2 processes together indicate that the energy bottleneck gets worse as a species’ brain size increases.

Third, immatures have relatively higher brain maintenance costs than adults, at least in mammals. Not only are juvenile mammals smaller and less experienced, but also they are more encephalized than adults because brain growth is completed before somatic growth [59,60,75]. This forces them to allocate a larger proportion of their energy budget on maintaining the brain (see [76] for humans). In addition, they face extra costs. The creation and pruning of numerous synaptic connections means that differentiating brains are more costly per unit weight than mature brains [46,77].

Finally, in both birds and mammals, proper brain development requires play, which is often quite vigorous and therefore energetically expensive. Indeed, primate species with more postnatal brain growth (and thus larger adult brains) play more [78]. We are not aware of similarly extensive comparisons in other mammals or birds (but see [79]).

**Fig 3:**
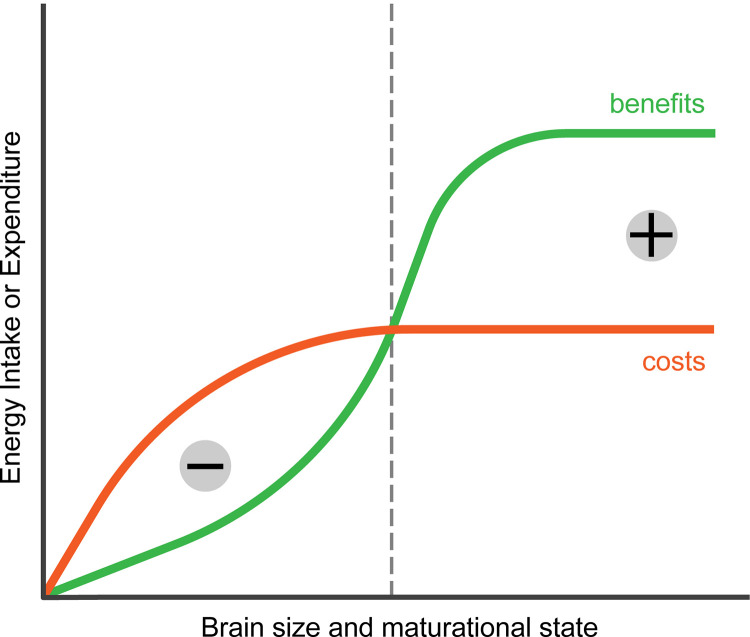
The bootstrapping problem for developing brains. In the absence of provisioning, immature animals developing their brains would likely face a long period of negative energy balance. Before the brain is fully grown and differentiated, it cannot provide adult-level cognitive benefits and the concomitant energy intake (green curve). The costs of growing, differentiating, and maintaining the brain (red curve) rise early and may even exceed adult values due to higher relative brain size of older immatures in mammals and costs of brain differentiation, before cognitive benefits, with their corresponding net energetic intake, stabilize at adult level. As a result, without parental provisioning the individual’s energy balance would be positive only after adulthood was reached.

These various obstacles to brain growth suggest that immature endothermic vertebrates, with their relatively large adult brain size [1], would face a seemingly insurmountable energy crunch if they were to grow and differentiate their brains with the energy they can obtain independently by themselves. This bootstrapping problem becomes more severe as relative brain size increases (Fig 3). It can be solved if parents donate the energy needed to grow and develop larger brains.

Endotherms have both extended parental provisioning and much larger brains than ectotherms. Here, we define parental provisioning as the total energetic investment into the young, directly (in eggs, through gestation, lactation or provisioning of food), or indirectly (by carrying or huddling to keep warm). In most ectothermic vertebrates, parental provisioning is limited and brief: they simply release (usually small: [80]) eggs. This is the likely ancestral state in vertebrates ([81]). The immatures of such species thus face these various developmental obstacles on their own. Consequently, these species face limits on brain size evolution, because larger brains would go hand in hand with unrealistically prolonged developmental periods. The evolution of extended parental provisioning accompanying the evolution of endothermy ([80,82]), therefore likely facilitated the subsequent evolution of larger brains size. The goal of this essay is to examine the idea that the evolution of extended parental provisioning has enabled species to overcome the brain’s bootstrapping problem and that brain size and parental provisioning have subsequently coevolved.

## The parental provisioning hypothesis

The parental provisioning hypothesis builds upon, yet greatly extends, Robert Martin’s [83] maternal energy hypothesis, which was initially based on a very different evolutionary logic, and perhaps because of this, failed to become widely adopted. It is instructive to trace the history of this hypothesis and how it gradually morphed into the parental provisioning hypothesis.

Martin [83] noted that the allometric scaling relationship with body size among placental mammals has the same exponent for both brain size and basal metabolic rate. This pattern suggested to him that “the resources channeled to the embryo from the mother” acted as a constraint on the brain size of a given species. The lower scaling exponent for brain size in birds and reptiles was attributed to their oviparity, and thus, consistent with this maternal energy effect. Initial attempts to test its predictions focusing on this allometric scaling were not favorable [84]. More direct tests were also not favorable. First, the precocial–altricial contrast in birds is inconsistent with this model. Precocial species, where young are well developed at birth and not provisioned after hatching, have smaller relative adult brain size but have much more developed brains at hatching than altricial ones, where young are poorly developed and need to be provisioned [85]. Second, maternal metabolic rate does not predict neonatal brain size or gestation length in a large sample of mammals [86] (but see [87] for a rebuttal).

These negative outcomes reduced the attention garnered by the maternal energy hypothesis, even though Martin [88] subsequently moved away from interspecific scaling. Focusing on placental mammals, he suggested that the pattern of correlations among “body size, brain size, basal metabolic rate, and gestation period indicates that the primary link is between maternal metabolic capacity and the developing brain of the offspring.” Thus, the hypothesis directly linked gestation length and maternal metabolic rate to neonatal brain size (cf. [89]). Perhaps the emphasis remained on gestation because Martin [83] had suggested that in primates, most brain growth is completed at birth. Although this may be correct for the number of neurons [90], neonatal brains in many species are less than half of adult size (e.g., [53,59]), especially in great apes and humans [91]. Moreover, brain differentiation (including myelination) is usually postnatal and among the most expensive aspects of brain development [46,77]. Thus, a proper test of the maternal energy hypothesis would require the inclusion of postnatal maternal investment in the form of lactation and (where relevant) provisioning.

Martin [88] also argued that the rate of maternal investment acts as a constraint on brain size, which, he suggested, leaves no room for variation in investment that produces adaptive variation in adult brain size (which would be achievable through variation in interbirth intervals or litter size). He argued that any links between a species’ brain size and ecology or social organization would be “a secondary consequence,” so that “there may be no very tight relationship between relative brain size and specific behavioral capacities.” Subsequent research has shown that adaptive explanations are supported for both the links with ecology [37,92], social organization [36], and cognitive performance [13,14]. This stance effectively reduced the appeal of the hypothesis.

Martin [87] later expanded the hypothesis’ scope by including lactation, and Martin and Isler [93] also considered of the overall duration of investment independent of metabolic turnover by the mother, reinforcing the conclusion that “development of the brain is heavily dependent on resources provided by the mother” ([87], p. 54). Unfortunately, these extensions garnered little attention.

Numerous comparative analyses have examined the link between adult or neonate brain size and life-history parameters in various groups, especially mammals (e.g., [29,30,54]; see also [32] for birds). Many studies found that larger-brained species take longer to reach adulthood (e.g., [34,53,55]). Although this points to competition between the growth of the brain and that of the body [94], consistent with the expensive brain hypothesis, such competition would also arise in the absence of extended parental provisioning and apparently can only be reduced by it. Therefore, this negative correlation in itself does not confirm the maternal energy hypothesis, although Barton and Capellini [55] could relate their findings to the maternal energy hypothesis, because “evolutionary changes in pre- and postnatal brain growth correlate specifically with duration of the relevant phases of maternal investment (gestation and lactation, respectively)” (see also [95]).

Note that the maternal energy hypothesis, after moving away from allometries, also reduced its stress on metabolic rates and began to cover the full period of parental investment to explain interspecific variation in brain size. The parental provisioning hypothesis expands it by including all forms of energetic investment and their rate and duration by both mothers, fathers, and helpers, and by regarding the process as an adaptive strategy (and not a constraint) to achieve the species’ optimum brain size. It also provides an explicit rationale for the need for extended parental provisioning.

### Testing a major assumption: Provisioning and brain growth rates

**Fig 4:**
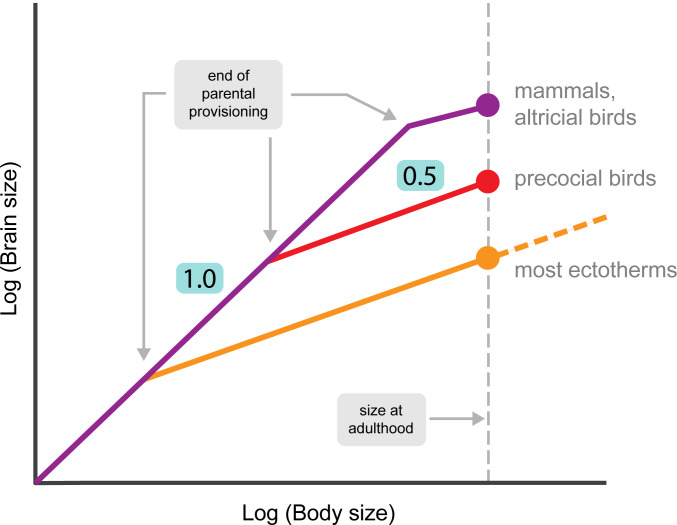
Schematic depiction of brain growth relative to body growth in different vertebrates as a function of parental provisioning. The first phase (parental provisioning) shows the same, steep slope (virtually isometric: 1.0). After the end of parental provisioning, the slope becomes very low (ca 0.2) in mammals and altricial birds, whereas it become intermediate in precocial birds and ectotherms (ca 0.5) until adulthood is reached, and in most of the latter continues at the same relative rate after that due to indeterminate growth (sources are provided in the main text).

The parental provisioning hypothesis is consistent with fundamental brain growth patterns (Fig 4). Across vertebrates, brain growth rates often show a sharp slowdown after a period of rapid growth [2,96,97], and this point coincides with the transition from parental provisioning to independence, i.e., self-sustained growth. In mammals, the initial period of rapid growth of the brain is generally isometric with that of the body [2,96,98]. In precocial species, born with relatively large brains [59], its growth slows down after birth, whereas in altricial mammals, growth continues to be high after birth [99]. In both precocial and altricial mammals, brain growth is completed by the end of parental provisioning, i.e., weaning [59,90,98], although subsequent differentiation may continue.

In birds, altricial and precocial species show different patterns of brain growth [85]. In altricials, brain growth is completed by the time offspring fledge [100,101], and thus entirely paid for by parental provisioning. Precocial birds face more of a bootstrapping problem, because there is little or no post-hatching provisioning and young must therefore find their own food. This explains why they have slower post-hatching brain growth than altricial species and achieve smaller relative brain size among adults (Fig 4).

In most ectothermic vertebrates, parental provisioning is far more limited. In most fishes, provisioning is entirely through eggs [102], and brain growth is high only during the very brief period before reserves in the egg are depleted and slows down soon after hatching [97]. However, since so much of the brain still needs to be developed, the brain growth trajectory remains steeper than for the endothermic vertebrates, as illustrated in Fig 4, and species with indeterminate growth retain the same slopes throughout life. The overall pattern is therefore consistent with the prediction that rates of brain growth are steep during the parental provisioning phase but clearly reduced thereafter.

## Testing the predictions of the parental provisioning hypothesis

Two important predictions follow from the hypothesis. First, we expect positive correlated evolution between extended parental provisioning and relative brain size, given that parents must be able to muster the energy to pay for their offspring’s brain growth. Second, we expect that this correlated evolution has made it possible for lineages with extended parental provisioning to evolve larger brains. Here, we examine the evidence for these predictions.

### Provisioning and brain size: Comparative tests

Comparative tests can assess the prediction that variation in the intensity and duration of parental provisioning shows correlated evolution with adult brain size. To start with birds, precocial and altricial species differ in brain size, with altricial species having larger brains for their body size than precocial species [85]. While long known [103], this difference has never been satisfactorily explained. The parental provisioning hypothesis links it to the amount of provisioning beyond egg size. In a study of 1,176 bird species, Griesser and colleagues [104] confirmed that the duration of parental provisioning showed strong correlated evolution with adult brain size.

Around 90% of bird species show biparental provisioning [105], and the modest variation in the number of caretakers is not correlated with relative brain size [104]. Among mammals, although over 80% of species have uniparental provisioning by the mother [106], allomaternal care (provisioning or carrying) is positively correlated with relative brain size, with the effect of male care being stronger than that of helpers [107], arguably because the male always helps whereas the number of helpers is highly variable and thus unreliable. These findings are therefore fully consistent with the parental provisioning hypothesis.

Turning to the ectotherm–endotherm contrast, extended parental provisioning may contribute to the explanation of the gap in relative brain size that separates them (Fig 1). In most ectotherms, provisioning stops at egg deposition of their (usually tiny [80]) eggs. In mammals, it only stops when offspring are weaned at roughly one third of adult size. Altricial birds fledge their young at close to adult size, whereas in precocial birds, although they do not provision young post-hatching, the eggs are large relative to those of ectotherms. Among ectotherms, cartilaginous fishes (Chondrichthyes: sharks, rays, skates, and sawfish) have brain sizes approaching those of endotherms [1]. Studies of chondrichthyes showed that species with matrotrophy, i.e., where young are supported beyond the yolk inside the egg, show larger relative brain size than those without it, at least for species up to 100 kg [108,109]. Although highly suggestive, the authors consider this support preliminary because the effect does not hold for the largest species. Lacking so far, are similar studies in the few radiations in ray-finned fishes (Actinopterygii), amphibians, and reptiles that show sufficient variation in parental provisioning. Overall, though, the existing studies of correlated evolution between extended parental provisioning and relative brain size overall support the parental provisioning hypothesis.

### Parental provisioning and the potential for encephalization

Interspecific brain–body allometries have long been explained as reflecting one major process, such as somatosensory needs or metabolic turnover [1,110], as artifacts of non-adaptive genetic correlations [111], or even as statistical artifacts [84]. However, none of these explanations is strongly supported [112,113]. Thus, the taxonomic variation in allometric slopes requires a new explanation, couched in terms of variable selective responses to new challenges by brain, body, or both.

The parental provisioning hypothesis may make a contribution to this debate. Its logic suggests that lineages with extended parental provisioning may more readily satisfy the preconditions for major evolutionary increases in brain size (encephalization) than those with limited and briefer parental provisioning, which may therefore remain caught in rather low-cognition niches. Marsh’s rule, which states that over evolutionary time species tend to become more encephalized (i.e., brains becoming larger relative to body size [1]), may therefore apply more strongly to lineages with more extended parental provisioning. Where this process is accompanied by enough adaptive variation in body size within a given lineage, this could produce steeper slopes of the brain–body relationship (where both are log-transformed) at higher taxonomic levels among extant species, a phenomenon known as the taxon-level effect [112].

One obvious way to test this prediction is to compare the slope of the brain–body allometry in precocial and altricial bird lineages. In precocial species, which do not provision their young beyond the resources provided in the egg, the bootstrapping problem may dampen selection on increased brain size, whereas those in altricial lineages have the opportunity to respond to it by increasing their provisioning. As a result, we would expect steeper slopes for the brain–body allometry among altricial birds than among precocial ones.

**Fig 5:**
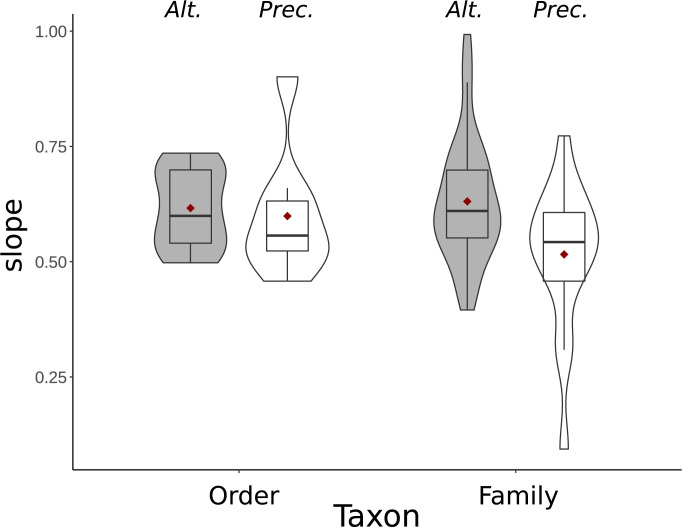
Brain–body allometries among altricial and precocial birds. Violin plots of slopes of the brain–body allometries of altricial (gray bars) and precocial (white bars) orders and families of birds. Data are taken from [104]. Orders or families were included when >5 species were available. Horizontal bars represent the median, red diamonds the mean, and boxes enclose the central 50% percentile range. The difference at the family level is significant (t = 2.60, DF = 26.46, *p* < 0.02). Sample sizes for altricial birds: 15 orders and 38 families; for precocial birds: 8 orders and 17 families.

Earlier results, produced for other purposes, provide a preliminary test. Nealen and Ricklefs [110] estimated the exponents of the brain–body allometry (i.e., the slopes of the log[brain]-log[body] regression) at multiple taxonomic levels. Their results revealed steeper slopes at the level of orders, families, and even genera among altricial taxa than among precocial ones. A more recent study [104] replicated this result with a modern phylogeny and a larger sample: A highly significant interaction effect between body weight and development mode on brain size revealed that altricial taxa have a far steeper slope. To visualize this effect, Fig 5 (data taken from [94]) shows the slope differences between altricial and precocial bird orders and families (based on ordinary least-squares regression).

An even more promising testing ground may be ectothermic vertebrates, which largely lack any post-hatching parental provisioning, even if some species guard young. Tsuboi and colleagues [97] reported that (phylogenetically corrected) brain–body allometry slopes at higher taxonomic levels are indeed clearly higher for birds (0.57) and mammals (0.59) than for fishes (0.50 for Actinopterygii and 0.41 for Chondrichthyes) and amphibians (0.46). Reptiles are closer to endotherms (0.56), but are better able than other ectotherms to maintain high body temperatures during activity through behavioral thermoregulation [114]. Tests at lower taxonomic levels have not been done yet. While these will no doubt soon emerge, this preliminary survey supports the proposition that lineages with extended parental provisioning are more likely to experience stronger encephalization, as expected under Marsh’s rule.

## Implications

Extending the maternal energy hypothesis, the parental provisioning hypothesis argues that brain size is not just limited by the ability of adults to avoid starvation, predation, and disease through cognitive means, but also by their cognitively supported ability to garner the time and energy to provide their young with the energy needed to construct the brains needed for this. Comparative work shows a strong correlation between total parental provisioning and brain size and also suggests that where extensive parental provisioning did not evolve, the evolutionary potential for greater encephalization is reduced. It thus attributes the ectotherm–endotherm gap in relative brain sizes partly to the evolution of systematic extended parental provisioning in early endotherms.

If we accept these conclusions, 2 important implications deserve attention. First, the parental provisioning hypothesis may also plug another gap in our understanding of brain size evolution, linked to immature survival. Second, the importance of parental provisioning for brain size also invites us to rethink the relationship between the cognitive abilities that produce fitness benefits and those that reduce fitness costs in selection on brain size.

### Parental provisioning and immature survival

The comparative tests reviewed above show that increased brain size tends to reduce reproductive rates and slows down development, which increases generation time. Even though it is unlikely that a species’ brain size is not adaptive, it may nonetheless be questioned whether the recorded increase in adult survival outweighs this dual fitness cost. We suggest that extended parental provisioning, and the concomitant continued protection of young, also provides another, previously overlooked adaptive advantage to larger brains.

There are currently no published comparative analyses of immature survival in relation to brain size. However, we recently conducted a preliminary analysis for primates, using published information on 18 species in 13 genera for which the relevant information from populations in undisturbed natural habitats has been published. We found that relative brain size improves survival until the age at first reproduction, in spite of the longer time needed to reach this point (unpublished results). Given the delay in the emergence of the various ecological skills produced by brains, it is difficult to imagine any other mechanism responsible for this remarkable pattern than parental provisioning and the associated protection.

Provided future work shows this result generalizes beyond primates, it supports the following evolutionary scenario for the evolution of parenting and its role in brain size evolution. Several forms of postnatal parental care or protection improve offspring survival [81] (and may also independently correlate with brain size [115]). Parental care may facilitate provisioning since this speeds up growth and thus reduces the time in the most vulnerable stage. Once parental provisioning beyond the egg has evolved, it alleviates the bootstrapping problem and provides reliable opportunities for practice and learning. These effects facilitate selection on increased brain size.

### Selection and brain size

**Fig 6:**
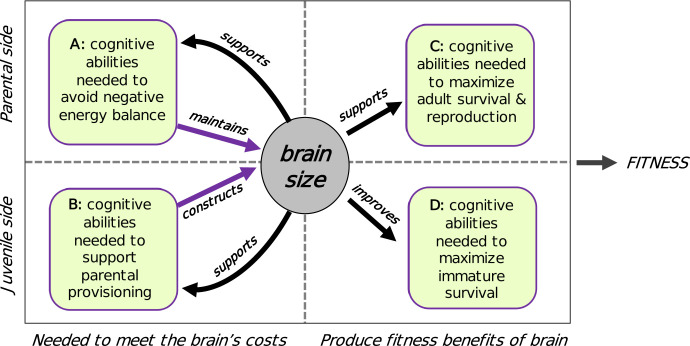
Brain size and the nature of cognition. Natural selection is expected to optimize brain size, by finding the optimum balance between the cognitive abilities (in the broad sense) required to pay for the costs of maintaining the adult brain (A) and constructing it during development (B) on the one hand, and the brain-size-dependent cognitive abilities that are translated into adult performance (C) and immature performance (survival: D) on the other hand. The 4 sets of cognitive abilities no doubt show high overlap, but their nature remains poorly studied. For birds, set A would presumably contain abilities such as migratory habits, food storing, extractive foraging, and communal roosting; set B abilities like predation avoidance (especially of nest contents), efficient foraging, habitat and nest site selection, flexibility, coordination ability; set C many of the same abilities as A and B, but also avoidance of predation on adults, post-independence skill learning, optimal mate choice, and social skills; and set D also nest site selection, nest building, and predation-sensitive provisioning.

The importance of parental provisioning for brain size also invites us to rethink how to integrate the various costs and benefits in selection on brain size. We can in principle recognize 4 sets of cognitive abilities, here defined broadly to also include sensory and motor abilities. A first set of abilities acts to maintain the adult brain (box A in Fig 6) by guaranteeing the stability of its energy supply. A second set enables adequate parental provisioning, and so serves to construct the adult brain (box B). The third and fourth set of cognitive abilities produce the cognitive performance that is responsible for the immature and adult survival as well as reproduction of its bearer (boxes C and D). Selection will favor an optimum brain size at which fitness is maximized. This optimum size depends on details of the ecological and social environment, the species’ bauplan, but also critically on the extent to which these 4 sets of cognitive skills overlap.

The cognitive abilities in A and B merely exist to maintain and build the brain, respectively. In the absence of the cognitive benefits produced by C and D, they would be futile. Thus, if the cognitive abilities in sets A and B are very different from those in C and D, selection on increased brain size would face a very high hurdle. This consideration therefore suggests strong overlap between them and that selection on brain size will be easier when cognitive abilities are not strictly domain specific, such as general intelligence and executive functions [16,22], because strictly domain-specific cognitive adaptations are less likely to enhance both the A-B and the C-D sets.

A recent analysis of birds [104] found that, once parental provisioning was controlled for, the correlations between brain size and the commonly measured indices of cognitive demand, such as group size, duration of social bonds, or ecological niches practically disappeared from the model, apart from ecological behaviors directly affecting energy balance, such as long-distance migration [116]. This suggests that the 2 sets of variables rely on the same cognitive abilities but parental provisioning is measured more precisely. Alternatively, some of the variables traditionally thought to affect brain size are perhaps not the main selective pressures, but feature in analyses merely because they happen to be available for many species.

The fact that the same cognitive abilities may serve to pay for energetic costs and produce direct fitness benefits raises a methodological problem. Most conventional methods for analyzing comparative data assume a unidirectional flow of causality from various variables representing fitness costs or benefits to the trait of interest. In the present case, depending on the stage of lineage evolution, brain size will be involved in a number of feedback loops (cf. Fig 6), and thus both respond to and drive the surrounding landscape of eco-social and life-history traits. Modeling evolutionary brain size trajectories that include such feedback loops will require new methods. These may include models for more robust and accurate estimation of shifts in the rate of change in variables across large phylogenies [117–119]. Likewise, we need models that allow for more accurate placement of variables as causes or effects in multivariate networks of traits, such as structural equation modeling or d-separation path analysis [120].

Promising insights into the evolution of brain sizes will also likely emerge from the ongoing re-evaluation of the importance of variation in comparative analyses: Methods focusing both on average patterns as well as the drivers of variance around trends (e.g., heteroscedasticity of brain–body size allometries observed across vertebrate taxa) are now able to incorporate phylogenetic relationships between species [121,122], providing new tools to disentangle the evolutionary history of brain sizes.

Finally, the parental provisioning hypothesis raises the broader question of which cognitive processes are the target of selection. It suggests that eco-cognitive and parenting skills have played a major role, with the fitness benefits of social bonds perhaps being derived from the abilities that evolved to enable parental provisioning, especially in lineages where more individuals coordinate parental activities [123].
